# Directional homing of glycosylation-modified bone marrow mesenchymal stem cells for bone defect repair

**DOI:** 10.1186/s12951-021-00969-3

**Published:** 2021-07-31

**Authors:** Long Chen, Wei Luo, Yuanzheng Wang, Xiongbo Song, Senlei Li, Jun Wu, Li Sun

**Affiliations:** 1 Department of Orthopedics, Guizhou Provincial People’s Hospital, Guiyang, 550000 Guizhou People’s Republic of China; 2 Guangdong Provincial Key Laboratory of Sensor Technology and Biomedical Instruments, School of Biomedical Engineering, Sun Yat-Sen University, Shenzhen, 518107 People’s Republic of China

**Keywords:** Bone marrow mesenchymal stem cells, Glycosylation, Directional homing, Bone defect repair

## Abstract

**Background:**

One of the greatest challenges for tissue-engineered bone is the low survival rate of locally grafted cells. The cell homing technology can effectively increase the number of these grafted cells, therefore, enhancing the repair of bone defects. Here we explore the effect of fucosylation modification on the directional homing of bone marrow mesenchymal stem cells (BMSCs) and their ability to repair bone defects.

**Results:**

Glycosylated BMSCs expressed high levels of the Sialyl Lewis-X (sLe^X^) antigen, which enabled the cells to efficiently bind to E- and P-selectins and to home to bone defect sites in vivo. Micro-CT and histological staining results confirmed that mice injected with FuT7-BMSCs showed an improved repair of bone defects compared to unmodified BMSCs.

**Conclusions:**

The glycosylation modification of BMSCs has significantly enhanced their directional homing ability to bone defect sites, therefore, promoting bone repair. Our results suggest that glycosylation-modified BMSCs can be used as the source of the cells for the tissue-engineered bone and provide a new approach for the treatment of bone defects.

**Graphic Abstract:**

**Figure:**
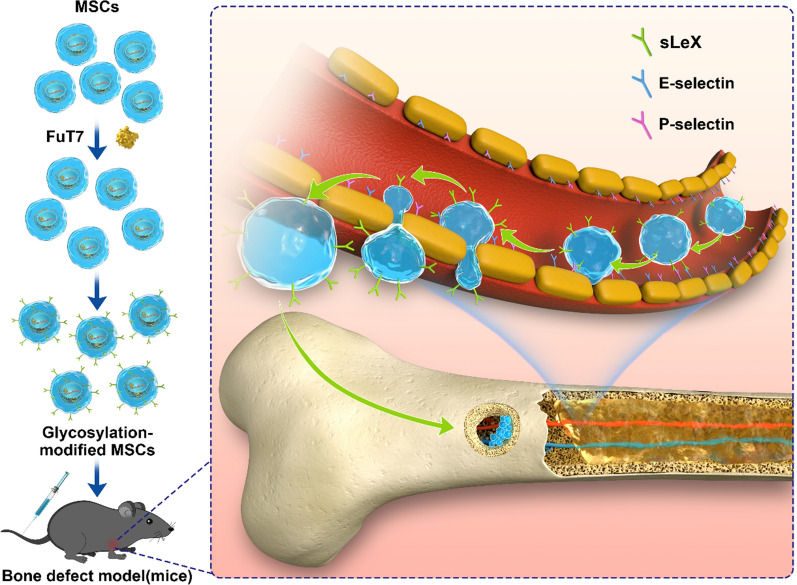

**Supplementary Information:**

The online version contains supplementary material available at 10.1186/s12951-021-00969-3.

## Background

Bone defect repair is a complex process regulated by numerous factors. It relies on the recruitment of cells with repair potential from the circulatory system or tissues surrounding the damaged area to the injury site. In response to damage microenvironment-induced cytokines and biomechanical factors, these cells will participate in the repair of bone defects either directly or indirectly. Mesenchymal stem cells (MSCs) are the ideal source of cells for tissue-engineered bones. Recent studies have shown that tissue-engineered bone generated from MSCs and biomaterials in vitro has a low survival rate after implantation into the sites of bone defects in vivo, and, therefore, is not the ideal repair option. Since MSCs lack the appropriate cell surface receptors involved in adhesion and homing, their homing efficiency from the circulatory system or surrounding tissues to the bone defect sites is also very low [1–4]. Therefore, increasing the number of local MSCs during bone repair in vivo has become an urgent problem in the field of tissue-engineered bone. The solution for this problem is also the key to the applications of MSCs in the treatment of bone-related diseases.

Recently, the delivery of MSCs to target damaged tissues and organs via directional homing to induce tissue repair have become a research hotspot in the field of regenerative medicine. Similar to the biological processes of tumor metastases spreading to various organs in the body with blood circulation, the directional homing of MSCs also requires the participation of multiple adhesion molecules. By leveraging the interactions between adhesion molecules and their ligands, the MSCs can overcome the blood flow shear force and can home to the target area via blood circulation to participate in the repair of damaged tissues. Several studies have shown that hematopoietic stem cells (HSPCs)—which are also derived from the mesoderm, similar to MSCs—can home to the bone marrow in large numbers in the presence or absence of body injuries [5–7]. However, HSPCs express the ligand structures of homing-related adhesion molecules on their surface, while the adhesion molecules E-selectin and P-selectin are widely expressed on the surface of bone marrow microvascular endothelial cells. As HSPCs reach the surface of microvascular endothelial cells in the bone marrow through blood circulation, the selectins will bind to their ligands one after another, enabling HSPCs to overcome the blood flow shear force. After tethering and rolling on the surface of endothelial cells, chemokines will activate the integrins VLA-4 and VLA-5 to induce adhesion and to promote strong attachment between cells. Finally, HSPCs will pass through the capillaries and enter the bone marrow. As cell adhesion molecules, E- and P- selectins are continuously expressed in capillaries and bone tissues. In response to inflammation or tumor invasion, endothelial cells at the injury site will be stimulated by interleukin-1(IL-1), tumor necrosis factor-alpha (TNF-α), and other inflammatory factors, leading to the upregulation of E- and P-selectin expression. This will assist the rolling and the adhesion of leukocytes to vascular endothelial cells under laminar flow conditions [8–10]. Among several ligands for selectins, sialylated Lewis oligosaccharide (sialyl-LewisX, or sLe^X^) is one of the smallest antigens that binds to selectins. The carbohydrates on the surface of some cell types are the main structures recognized by selectins and can efficiently mediate the selectin/ligand binding [11–13]. Based on these findings, we hypothesized that increasing the expression of sLe^X^ antigen on the surface of MSCs will improve their adhesion to selectins, therefore, allowing the directional homing of MSCs.

HPSCs express CD44, a proteoglycan with galactose linked to the α-2,3-sialic acid group, and N-acetylglucosamine linked to the α-1,3-fucose group, forming a sialylated sLe^X^ structure. Although MSCs have naturally low levels of the sLe^X^ antigen, they express another type of another CD44 isoform, with N-acetylglucosamine that lacks α-1,3-fucose modification [14, 15]. Fucosyltransferase VII (FuT7), an important tool in protein glycosylation engineering, can catalyze the transfer of fucose residues from GDP-fucose to the receptor and promote the formation of the corresponding glycosidic bond. Based on the structural characteristics of MSC surface receptors and the theory of protein glycosylation, here we propose to attach the α-1,3-fucose group to the N-acetylglucosamine in MSCs by inducting the expression of exogenous genes. As a result, new glycoprotein epitopes will be formed and will generate many sLe^X^ antigens on the surface of MSCs, enhancing their directional homing ability.

Bone defects caused by trauma, infection, or tumors are clinically very common. The current generally accepted treatment of bone defects is bone transplantation. Bone grafts mainly include autologous, allogeneic, and xenogeneic bone; however, this type of treatment has several limitations, such as limited sources, trauma to the donor site, immunologic rejection, disease transmission, and so on. With the rapid development of materials science and tissue engineering technologies over the past two decades, researchers have generated numerous bone replacement materials using novel methods and approaches. However, the low survival rate of locally grafted cells has resulted in poor internal vascularization of the replacement material and insufficient biological functions, restricting their clinical application. The emergence of cell homing technology provided a new opportunity for the artificial bone replacement materials to repair bone defects. Here in this study we transfected the FuT7 gene into MSCs using lentiviral transfection, to attach the α-1,3-fucose group to the N-acetylglucosamine of the CD44 molecule on the surface of MSCs, resulting in the generation of a large number of sLe^X^ antigens on cell surfaces. Furthermore, in vitro and in vivo experiments were conducted to explore the effect of glycosylation on the directional homing ability of MSCs, and on repair of bone defects after MSCs homing. These findings will provide a foundation for the application of glycosylation-modified MSCs to repair bone defects.

## Results

### Isolation, extraction, and identification of bone marrow mesenchymal stem cells (BMSCs)

**Fig. 1:**
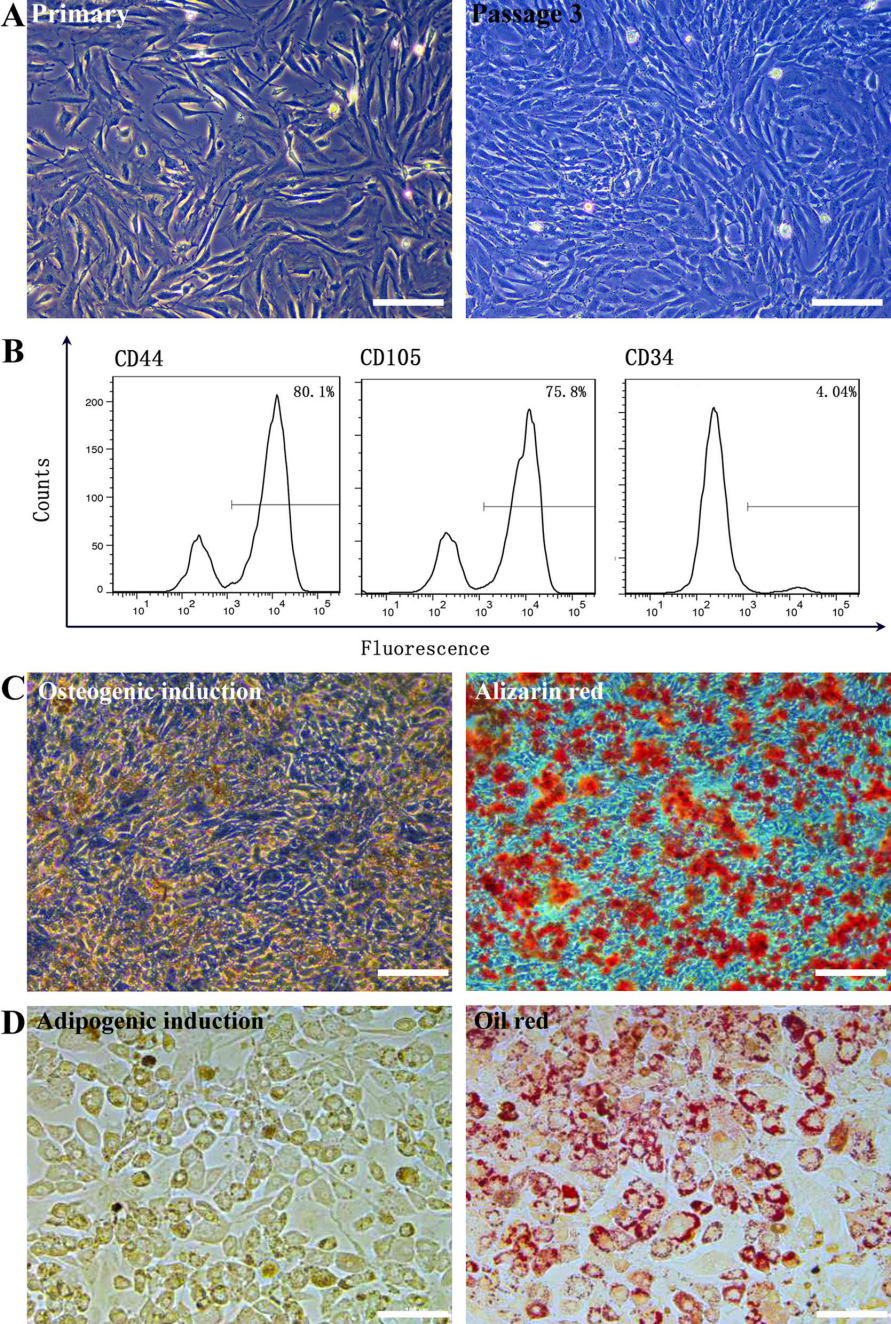
Identification of BMSCs. Representative images showing **A** morphology of BMSCs, scale bar = 100 μm; **B** Expression of CD44, CD105 and CD34 using flow cytometry; **C** alizarin red staining (osteogenesis) at day 21, scale bar = 100 μm; **D** Oil Red O staining (adipogenesis) at day 14, scale bar = 100 μm

Primary BMSCs had short fusiform (or spindle-shaped) morphology. After culturing for 6 d, the cells reached more than 70% confluence, and the rate of cell proliferation increased. When the cell confluence reached over 80%, cells were subcultured. After passaging, the cell morphology started to change into a long spindle-like shape, showing typical “fish-like” and “vortex-like” arrangements (Fig. 1A). Flow cytometry was used to identify cell surface markers. Among them, CD44 and CD105, cell stemness markers, accounted for 83.8 and 83.5%, respectively, while CD34, the endothelial marker, accounted for only 0.336% (Fig. 1B). These results are consistent with the percentages reported in the literature, indicating that cells isolated and cultured in this study were indeed MSCs. Next, the cells were induced using osteogenic and adipogenic media and then stained with Alizarin Red and Oil Red O, respectively. Results showed that both sets of cultures were Alizarin Red and Oil Red O staining positive, respectively, indicating that these cells possessed both osteogenic and adipogenic differentiation potentials (Fig. 1C and D).

### Surface glycosylation modification of BMSCs

**Fig. 2:**
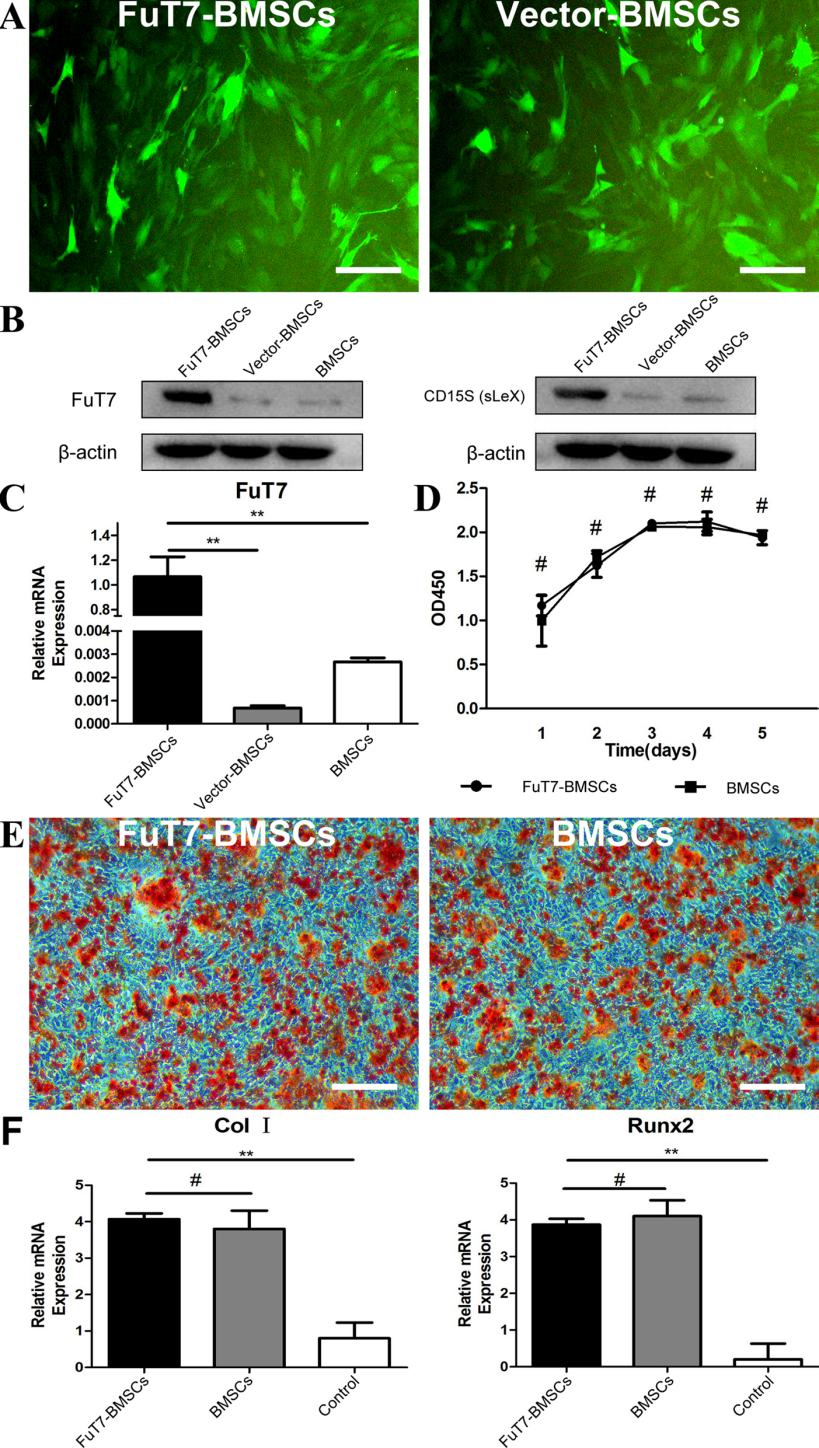
Surface glycosylation modification of BMSCs. Representative images presenting **A** FuT7 and empty vector expression in BMSCs, scale bar = 100 μm; **B** FuT7 and sLeX (CD15S) for surface glycosylation modification of BMSCs by western blotting; **C** relative gene expression of FuT7 for surface glycosylation modification of BMSCs, ^**^p < 0.01; **D** cell proliferation analysis as shown by CCK-8 for FuT7-BMSCs and BMSCs, ^#^*p* > 0.05; **E** alizarin red staining (osteogenesis) at day 21 for FuT7-BMSCs and BMSCs, scale bar = 200 μm; **F** relative gene expression of Col I and Runx2 for FuT7-BMSCs and BMSCs, ^**^p < 0.01, ^#^*p* > 0.05

After transfecting the FuT7 lentiviral vectors into cells, both FuT7- and the control (empty vector)-containing cells expressed GFP, confirming the successful transfection of BMSCs and allowing us to observe green fluorescence under the fluorescence microscope (Fig. 2A). Western Blotting showed that the glycosylated BMSCs expressed higher levels of FuT7 and sLe^X^ (CD15S) proteins compared to the empty vector BMSCs and unglycosylated BMSCs (Fig. 2B). The RT-PCR demonstrated that glycosylated BMSCs expressed higher gene expression levels of *FuT7* compared to empty vector BMSCs and unglycosylated BMSCs (Fig. 2C). The CCK-8 assay showed that glycosylation did not have any effect on the proliferation of MSCs (*p* > 0.05) (Fig. 2D). To evaluate whether glycosylation affected the osteogenic ability of these cells, cultures were assessed by Alizarin Red staining and by PCR. Alizarin red staining allowed us to detect the formation of mineralized nodules by either glycosylated BMSCs or untreated BMSCs. Our results demonstrated that both groups displayed good osteogenic differentiation potential and glycosylation did not have an effect on mineralized nodule formation (Fig. 2E). The real-time fluorescent PCR confirmed that there were no significant differences in the expression of osteogenic genes *Col1* and *Runx2* (*p* > 0.05) between glycosylated BMSCs and untreated BMSCs (Fig. 2F).

**Fig. 3:**
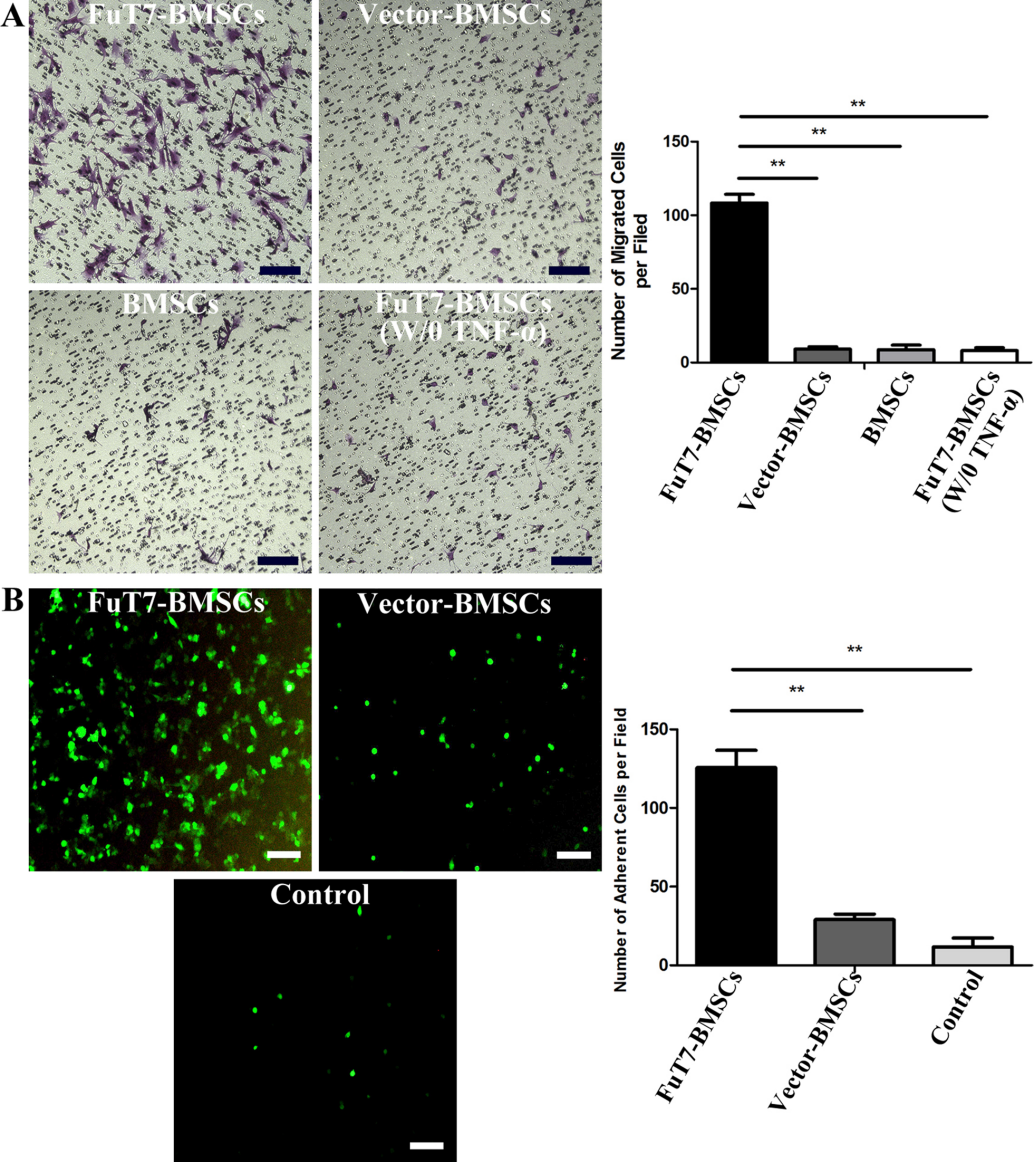
The migration and static cell adhesion abilities of glycosylated BMSCs. Representative images presenting **A** Transwell cell migration assay for glycosylated BMSCs, scale bar = 200 μm, ^**^p < 0.05; **B** The static adhesion abilities of glycosylated BMSCs, scale bar = 100 μm, ^**^p < 0.05

**Fig. 4:**
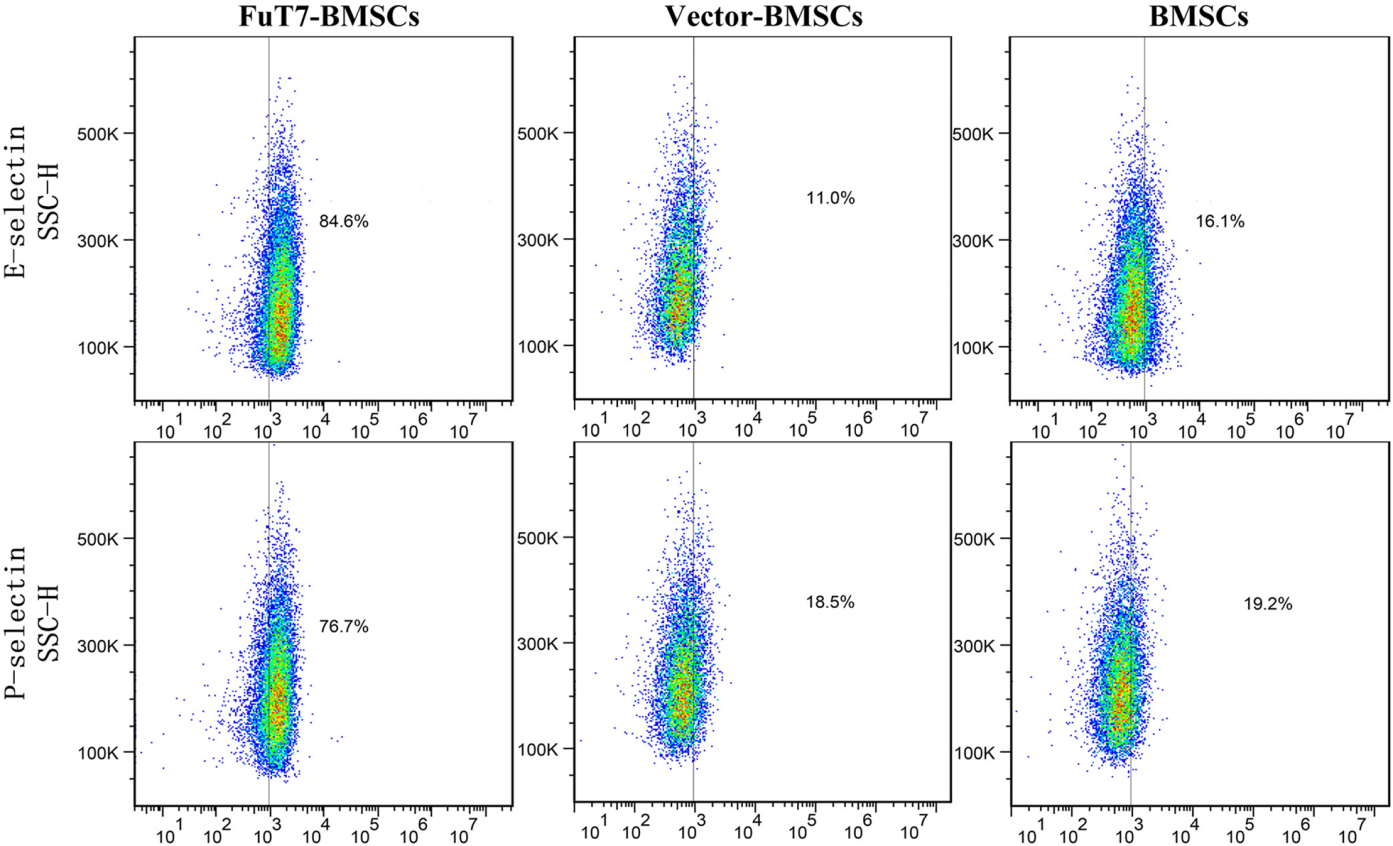
The binding capacities of glycosylated BMSCs to E- and P- selectins detected by flow cytometry

Transwell cell migration assay indicated that TNF-α stimulated MMECs to produce E- and P-selectins, inducing the migration of glycosylated BMSCs. Furthermore, this migration ability of BMSCs was significantly improved by glycosylation (*p* < 0.05) (Fig. 3A). The static adhesion abilities of glycosylated BMSCs and MMECs were evaluated by the flushing strength of PBS. Our results showed that the static cell adhesion abilities of glycosylated BMSCs to MMECs were significantly higher compared to the control group or the negative control group (*p* < 0.05) (Fig. 3B). The binding capacities of glycosylated BMSCs to E- and P- selectins were also detected by flow cytometry. Our results demonstrated that the binding capacity of glycosylated BMSCs to E- and P- selectins was stronger than of the control group or the blank group: the binding capacity to E-selectin was 84.6%, and the binding capacity to P-selectin was 76.7% (Fig. 4).

### Detection of the directional homing ability of glycosylated BMSCs using bone defect mouse model

**Fig. 5:**
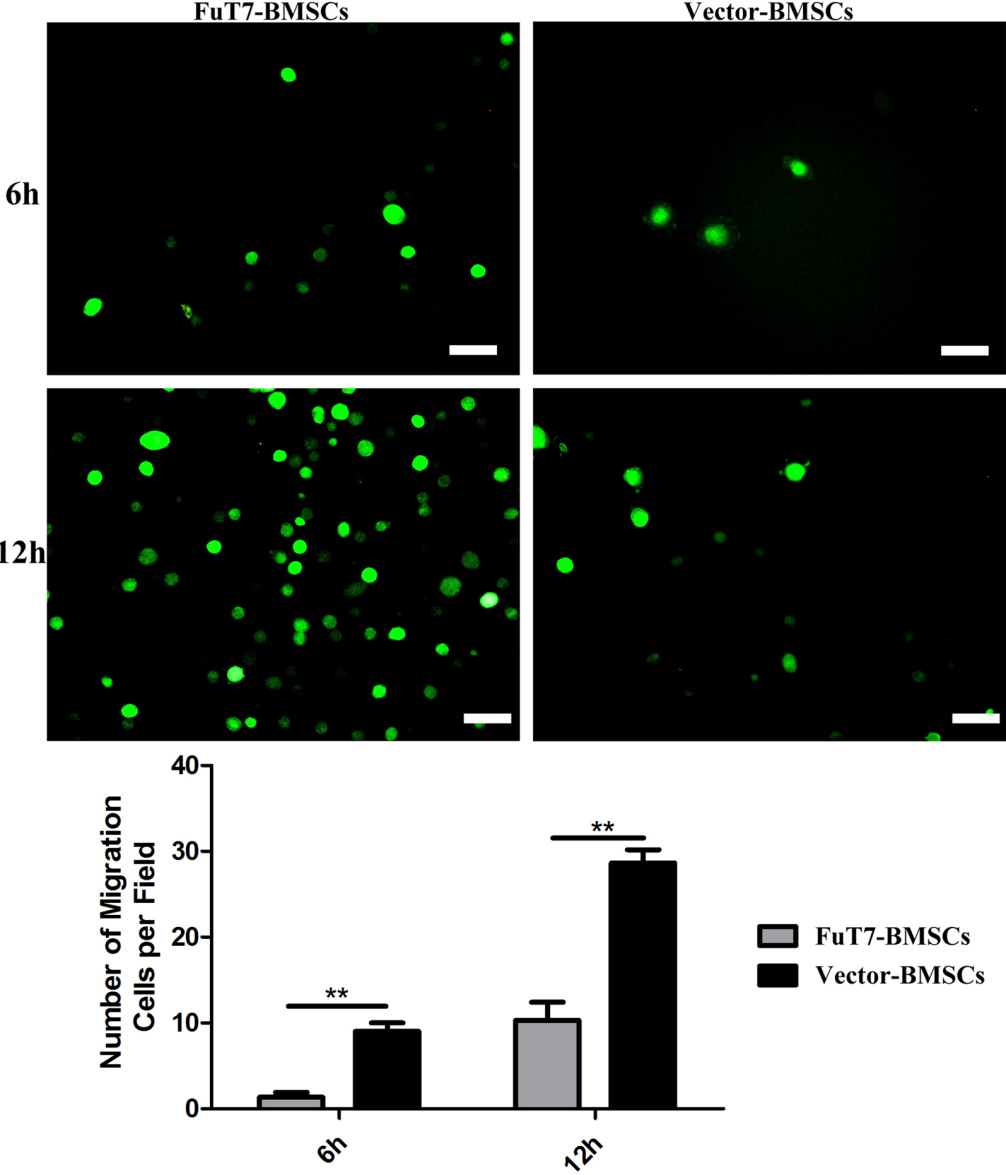
The bone marrow flushing method for detection of the directional homing ability of glycosylated BMSCs, scale bar = 200 μm, ^**^p < 0.05

**Fig. 6:**
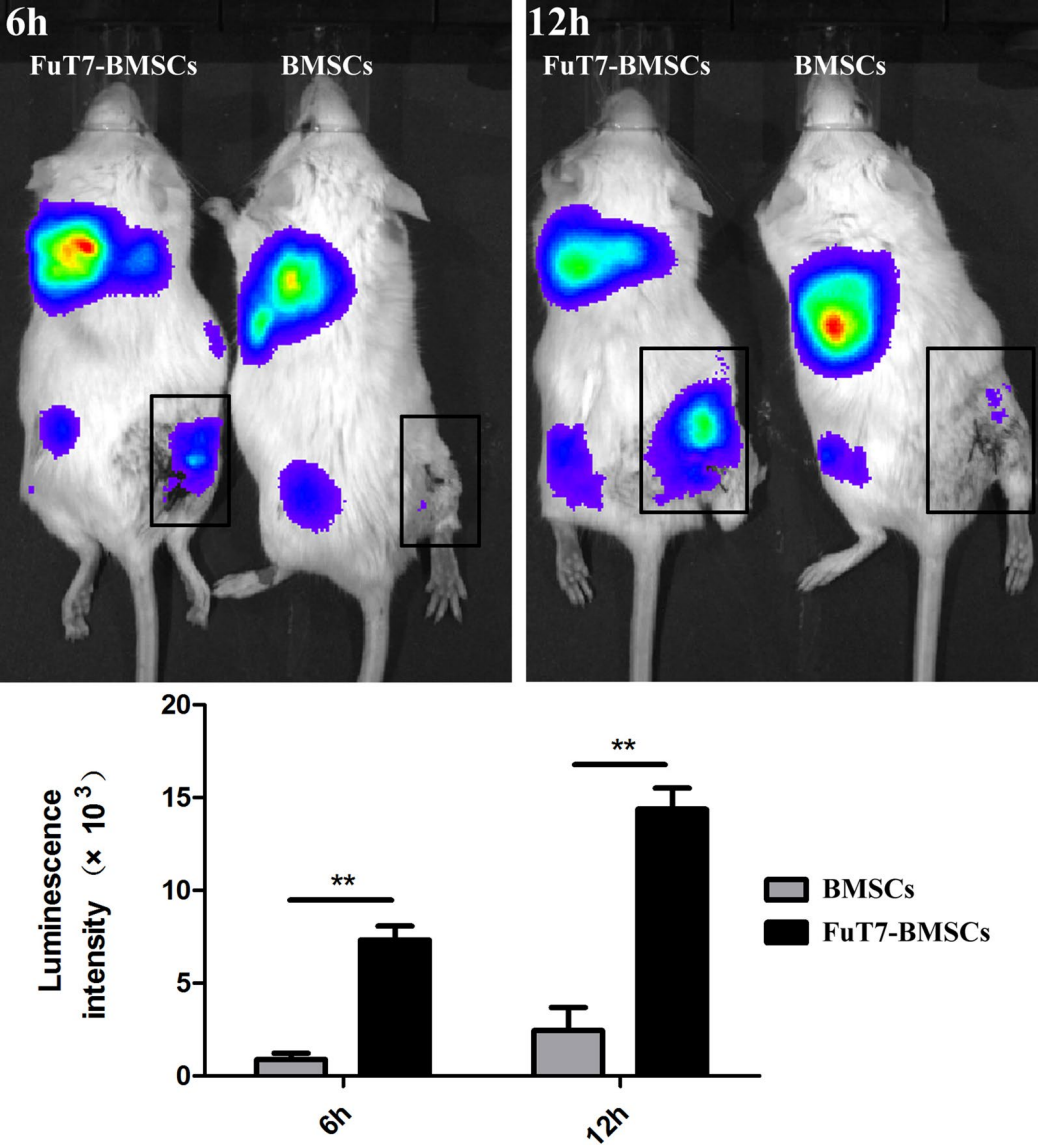
The in vivo imaging method for detection of the directional homing ability of glycosylated BMSCs, ^**^p < 0.05

**Fig. 7:**
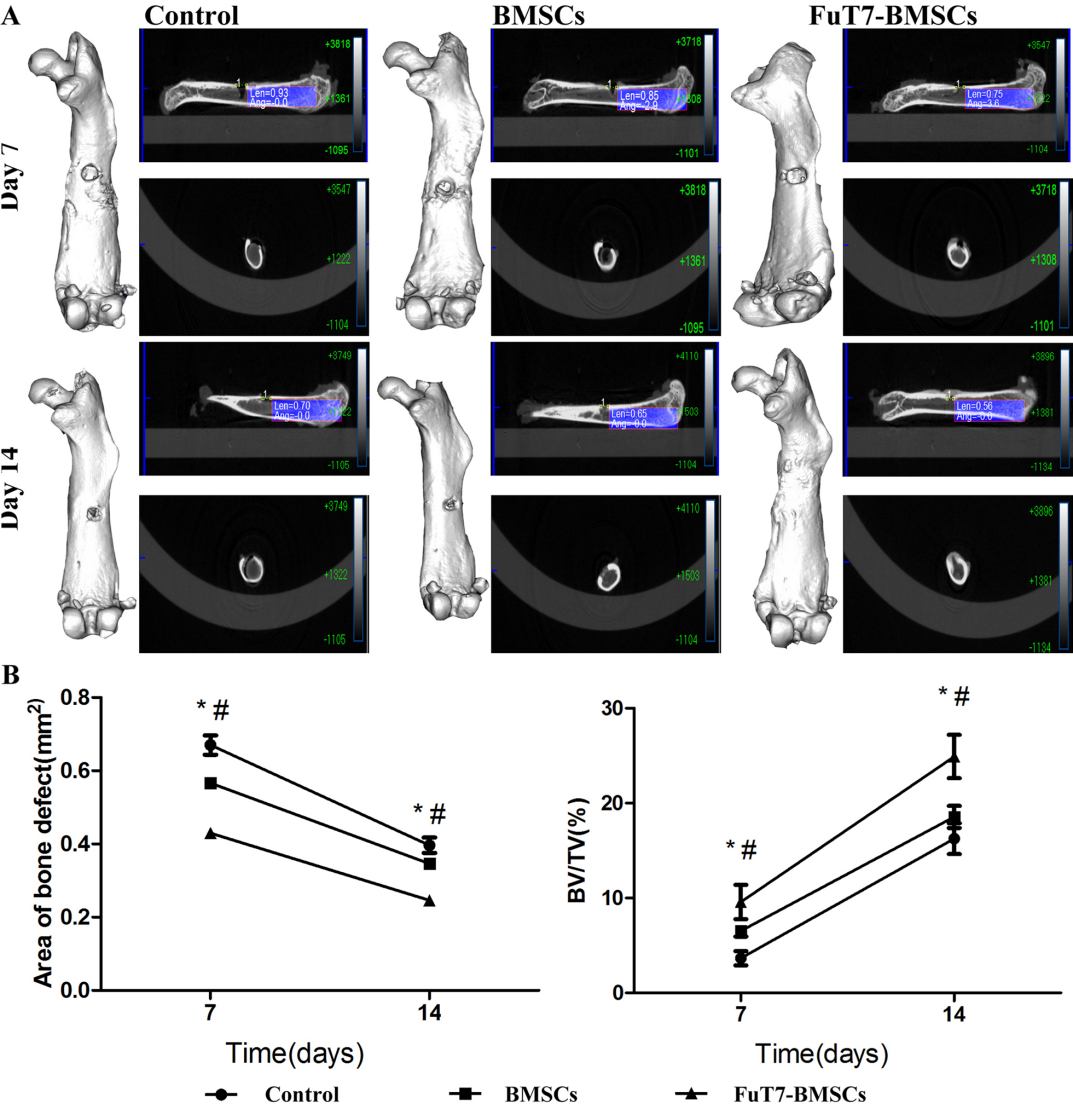
The result of Micro-CT for regenerated bone in femoral bone defect mouse model at 7 and 14 days. Representative images presenting **A** Post-injection micro-CT images for regenerated bone in femoral bone defect mouse model at 7 and 14 days; **B** The area of bone defect for femoral bone defect mouse model at 7 and 14 days after injection, ^*^FuT7-BMSCs versus BMSCs p < 0.05, ^#^FuT7-BMSCs vs Control p < 0.05; (C) Bone volume (BV)/Tissue volume (TV) ratio for femoral bone defect mouse model at 7 and 14 days after injection, ^*^FuT7-BMSCs versus BMSCs p < 0.05, ^#^FuT7-BMSCs vs Control p < 0.05

**Fig. 8:**
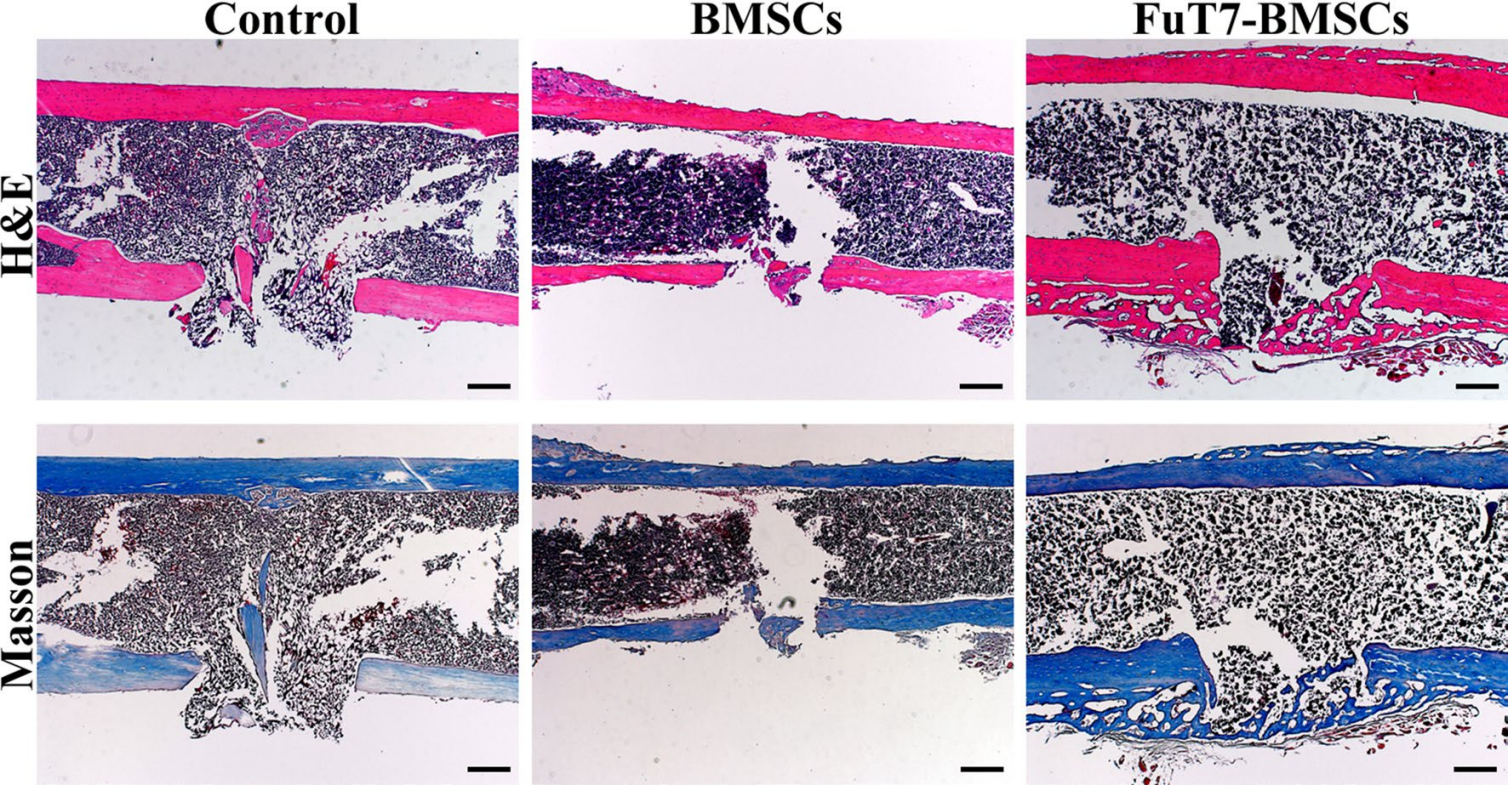
The H&E and Masson staining for regenerated bone in femoral bone defect mouse model at 10 days, scale bar = 200 μm

The results of the bone marrow flushing method suggested that glycosylation treatment significantly improved the homing ability of BMSCs to the bone defect site in vivo (*p* < 0.05) (Fig. 5). To further explore the directional homing ability of glycosylated BMSCs in the mouse model of bone defect, the in vivo imaging method was used. Results showed that glycosylation significantly improved the directional homing ability of BMSCs to reach the bone defect site in our mouse model (*p* < 0.05) (Fig. 6). Using the 3D femur reconstruction and the cross-sectional scan of the femoral sagittal axis, the Micro-CT results suggested that at days 7 and 14, the glycosylated BMSCs group had smaller defect repair area and higher BV/TV ratio (*p* < 0.05), indicating better bone repair, compared to the other two groups (Fig. 7). The H&E and Masson staining confirmed that the glycosylated BMSCs group showed better bone defect repair properties, since the trabecular bone and the dark blue calcium nodules were more apparent than in the other two groups. Furthermore, the thin layer of callus had covered almost the entire bone defect area in glycosylated BMSCs group (Fig. 8).

## Discussion

In 1983, Gallatin et al. reported for the first time that lymphocytes circulating in the blood could selectively enter secondary lymphoid organs, naming this process as lymphocyte “homing” [16]. Since then, “homing” has been used to describe the process during which cells (especially white blood cells) migrate directionally towards and colonize specific target tissues. In recent years, with the rise of stem cell therapy, this concept has been extended to a variety of cells, including MSCs. The directional homing of MSCs refers to the process, driven by various factors, during which autologous or exogenous MSCs migrate through the vascular endothelium to target tissues or organs, and colonize. MSCs possess multidirectional differentiation potential and the ability to maintain body homeostasis, which are the keys to their capabilities in promoting tissue repair and regeneration. When local tissues are subjected to severe and irreversible injuries, MSCs are often required to home to the injury sites in order to provide necessary cells to initiate tissue repair [17]. Since MSCs lack cell surface antigens related to adhesion and homing, their homing efficiency to migrate from the circulatory system or surrounding tissues to the injury site is relatively low. With the continuous development of tissue engineering, various stem cell technologies have been applied to enhance the homing ability of MSCs, thereby providing new methodologies for the repair of various tissue and organs. For bone tissue engineering, stem cell homing based on scaffold features and cell modification are most likely to enhance the homing ability of MSCs. Scaffold is one of the three key elements for bone tissue engineering, and it can be designed and modified by chemical and physical methods to recruit MSCs to reach the bone defect area. For cell modification, it mainly includes three technologies to enhance the homing ability of MSCs: magnetic guidance, genetic modification and cell surface engineering.

The homing process of MSCs can be divided into five main steps: tethering/rolling, activation, arrest, transmigration, and migration. For these five homing steps, the strategies to enhance the homing ability of MSCs include: (1) magnetic guidance; (2) gene modification; (3) cell surface modification; (4) priming; and (5) modification of target tissues [18]. The cell surfaces of almost all organisms are covered with different types of sugar molecules. Glycosylation is a process that modifies the sugar chains on the protein surface via enzymatic reactions, leading to changes in protein function [19]. In this study, a lentiviral vector carrying a FuT7 gene was constructed, and then transfected into MSCs to link the α-1,3-fucose group to the N-acetylglucosamine of the CD44 molecule on the surface of MSCs, therefore, transforming them into cells with a strong affinity for selectins. After the cells were stably transfected, Western Blotting and RT-PCR confirmed that glycosylated MSCs expressed high levels of FuT7, both on protein and gene levels. Western Blotting was also used to confirm the high expression of the sLe^X^ antigen. Using flow cytometry, static adhesion, and Transwell migration experiments, we demonstrated that the glycosylated MSCs displayed stronger adhesion and chemotaxis to E- and P-selectins, supporting our hypothesis. To further verify the directional homing effect of glycosylated MSCs in vivo, we performed the bone marrow flushing and in vivo imaging experiments using the drill-hole bone defect mouse model. MSCs labeled with green fluorescence (GFP) and firefly fluorescence were injected into mice with bone defects through the tail vein. The number of homing cells was measured at 6 and 12 h, respectively. These two methods further confirmed that glycosylation enhanced the directional homing ability of MSCs. Other methods, such as chemical modification or genetic modification, to enhance the directional homing ability of cells have also been reported [20–22]. Although the homing ability of cells to the injured site could be enhanced, these strategies often have several limitations, such as complex modification methods, poor cell stability after modification, damage to the original biological functions of the cells, and potential carcinogenic risk. Therefore, in terms of safety and feasibility, we believe that the cell glycosylation modification method is more advantageous compared to the other methods.

The bone repair process usually undergoes several specific stages: inflammatory hematoma stage, soft callus formation stage, hard callus formation stage, and callus remodeling stage. In the inflammatory stage, a hematoma usually occurs within 7 days after injury. During that stage, a large number of inflammatory cells and inflammatory factors (such as IL-1, TNF-α, among others) is accumulated at the defect site. These inflammatory factors will stimulate vascular endothelial cells to express high levels of selectins. Around day 14 after bone injury, the repair process will progress to the soft callus formation stage. Since the formation mechanism of the long bone is endochondral ossification, large numbers of chondrocytes will begin to appear and will be continuously replaced by osteoblasts. Based on the Micro-CT scans of the femur defect at days 7 and 14, we observed that the glycosylated MSCs group had a smaller defect area and higher BV/TV ratio. Furthermore, the histological staining showed that in the glycosylated MSCs group, chondrocytes were congregating around the bone defect, and a considerable amount of callus was observed covering the defect surface during the early stage. To further explore whether the surface glycosylation modification of MSCs can affect their osteogenic ability, osteogenic differentiation experiments were performed in vitro. Based on the number of calcified nodules (Alizarin Red staining) and the expression levels of *Col1* and *Runx2*, there was no significant difference in osteogenic potential between surface glycosylation-modified MSCs and untreated MSCs. Furthermore, the CCK-8 assay verified that glycosylation modification had no significant effect on the proliferation of MSCs. Collectively, our results suggest that in vivo, after the modified cells reached the bone defect site via the blood circulation, they colonized the endosteum surface, while maintaining their self-proliferation abilities and osteogenic differentiation. Our findings are also consistent with the research reported by Sackstein et al. [14, 15]. Therefore, it is possible that the significantly enhanced bone repair observed in the glycosylation group could be attributed to the increased migration of MSCs to the defect site. These MSCs would produce cytokines that promote bone repair, further stimulating bone healing in the glycosylation group.

## Conclusions

In this study, the in vitro glycosylation modification was used to generate sLe^X^ antigens on the surface of MSCs, leading to significantly enhanced binding to E- and P-selectins. At the same time, this modification did not affect MSC proliferation and osteogenic differentiation potential. In the mouse model of bone defect, the glycosylation modification of MSCs significantly improved the directional homing ability of the cells to the bone defect site, resulting in increased number of homing cells and enhanced bone repair. Therefore, glycosylated MSCs can be utilized as the preferred source of cells for tissue-engineered bone, providing a novel approach for treating bone defects, and have broad application potential.

## Methods

### Isolation, extraction, and identification of BMSCs

Six 4-week-old male SPF FVB/N mice were sacrificed by cervical dislocation and then placed into 75% ethanol solution for 5 min. The bilateral tibia and femur of the mice were dissected out using sterilized surgical instruments in the laminar flow hood. The metaphyses on both sides were cut off using ophthalmic scissors, and a 2-mL syringe containing PBS was used to flush the bone marrow from the long diaphysis into a 15-mL centrifuge tube. The centrifuge tube was then centrifuged at 500 × *g* for 15 min, the supernatant was discarded, and the cell pellets were resuspended in PBS. The cell concentration was adjusted to 1 × 10^8^ cells/mL to prepare the single cell suspension, and then separated using density gradient centrifugation.

The confluent passage P3 cells were collected and the expression of CD44 (eBioscience, CA, USA), CD105 (eBioscience, CA, USA), and CD34 (eBioscience, CA, USA) was detected using flow cytometry. The confluent passage P3 cells were cultured either in osteogenic induction medium or in adipogenic induction medium (Cyagen Biosciences Inc, CA, USA) for 21 or 14 days, respectively. Next, cultures were stained with either Alizarin Red or Oil Red O (Cyagen Biosciences Inc, CA, USA) to verify their osteogenic or adipogenic differentiation potential, respectively.

### Surface glycosylation modification of BMSCs

The pLVX-IRES-ZsGreen 1-PGK-Puro plasmid (1 μg) (Genechem, Shanghai, China), DMEM (1 ml) (Hyclone, CA, USA), Lenti-HG Mix (10 μL), and HG transgene reagent (60 μL) were added into the petri dish to co-transfect the 293 T cells. After 12 h of transfection, 100 × Enhancing buffer was added drop-by-drop to promote transfection. After 20 h of transfection, the medium in the petri dish was replaced. Next, 48 h after replacing the medium, the cell supernatant was transferred to a 50-ml centrifuge tube and centrifuged at 4500 × g for 5 min. After that, the supernatant was filtered using a 0.45-μm filter and transferred to a new centrifuge tube. The filtrate was then concentrated in batches. The final centrifugation was performed at 4500 × g for 20 min, and the liquid in the upper layer of the filter was the viral concentrate.

The DMEM/F12 complete medium was used to prepare a cell suspension at a density of 3 × 10^4^ cells/mL, and 100 μL of the suspension was pipetted into a 96-well plate. Based on our preliminary experiments, the corresponding amount of virus (Genechem, Shanghai, China) was added, MOI = (virus titer × virus volume)/number of cells; Polybrene (Sigma, Saint Louis, USA) (10 μg/mL) was also added to screen for infected cells. The cells were cultured for another 2–3 days to maintain cell viability. Cell viability was observed using an inverted microscope. The medium was replaced every day to remove the dead cells. The infection efficiency was observed using a fluorescence microscope, with the green fluorescence indicating successfully infected cells. The same method was utilized to transfect BMSCs with the control (empty vector) virus.

The FuT7-BMSCs, Vector-BMSCs, and untreated BMSCs were washed with PBS, and 200 μL of RIPA protein lysis solution containing PMSF (Sigma, Saint Louis, USA) was added to each 25-cm^2^ culture flask. Cells were scraped, transferred into the microcentrifuge tube, and lysed on ice for 30 min. The lysates were then centrifuged at 12,000 × g for 10 min at 4 °C. The FuT7 and sLe^X^ (Antibody, Abcam, MA, USA) concentration of the supernatant was measured, SDS-PAGE gel electrophoresis (Beyotime Biotechnology, Shanghai, China) was performed, followed by membrane transfer, antibody incubation, exposure, and analysis.

FuT7-BMSCs, Vector-BMSCs, and untreated BMSCs were washed with PBS. The Trizol reagent was used to extract the intracellular RNA, and a reverse transcription kit (Takara Bio Inc, Japan) was used for reverse transcription. The SYBR® Premix Ex Taq II kit (Takara Bio Inc, Japan) was used to evaluate FuT7 gene expression by the IQ5 real-time PCR system. The primer sequences are listed in Table 1.

**Table 1:** Primer sequences (5'-3') used for RT-PCR

Gene	Forward	Reverse
FuT7	ATGGAATCGCCCAGTAATACCC	TTTGGCCGGTAGTGGGGAT
β-actin	AACAGTCCGCCTAGAAGCAC	CGTTGACATCCGTAAAGACC

The passage P3 FuT7-BMSCs and BMSCs were used in these experiments. After trypsin digestion, cells were seeded into 96-well plates at 1 × 10^5^ cells/mL, 100 μL/well. Five groups of cells corresponding to day 1, 2, 3, 4, and 5 were prepared. The 96-well plates from the corresponding number of days were taken out and 10 μL of CCK reagent (MedChemExpress, NJ, USA) was added to each well according to the CCK-8 manual. Next, the 96-well plates were incubated in a 37℃ incubator for 2 h. Optical density of each well was measured using a microplate reader at a wavelength of 450 nm.

The mouse microvascular endothelial cell (MMEC) (MT Bio Inc, Shanghai, China) suspension (4 × 10^4^ cells/mL) was pipetted into 24-well plates, at 600 μL per well. Next, to stimulate the MMECs, TNF-α (10 ng/mL) (Sino Biological Inc, Beijing, China) was added to each well of the culture plates and the plates were placed into the cell culture incubator for 6 h. The Transwell inserts were placed into the 24-well plates, 5 × 10^3^ cells/mL of different BMSCs were added to each insert (Table 2, Additional file 1: Figure S1), and cells were then incubated in a 37 °C incubator. After 12 h of incubation, the Transwell inserts for each group were taken out and washed three times with PBS. The cells were fixed with 4% paraformaldehyde for 30 min and then washed three times with PBS. The cells on the Transwell insert membrane were stained with crystal violet solution (500 μL per well) for 30 min. The top layer of the insert membrane was washed under running water to remove the dye while avoiding touching the insert bottom. The inside of the insert was dried using a cotton swab, and the cells were observed, imaged, and counted under a microscope.

**Table 2:** Groups of the in vitro migration experiment of glycosylated BMSCs

Group	Upper insert	Lower insert
Group 1	FuT7-BMSCs	MMECs + TNF-α
Group 2	BMSCs	MMECs + TNF-α
Group 3	FuT7-BMSCs	MMECs
Group 4	Vector-BMSCs (empty vector)	MMECs + TNF-α

The passage P3 MMECs were seeded into 96-well plates in the complete medium containing 25 ng/mL TNF-α. The plates were incubated at 37 °C and 5% CO_2_ for 6 h; after that, the medium was removed. The BMSCs from each group were prepared as single-cell suspensions, added to each well of the 96-well plates at 1 × 10^3^ cells/well, and the plates were incubated for 1 h. Next, the plates were taken out, the culture medium was aspirated and discarded, and the wells were washed three times with PBS. The cells were then observed, imaged, and counted under a fluorescent microscope. (Three random visual fields were selected for each group; the empty virus transfection group was used as the control group, and the group without TNF-α stimulation was used as the negative control group).

The BMSCs were divided into three groups: FuT7 group, empty vector transfection group (Vector), and untreated group. These three groups of BMSCs were resuspended in 1% BSA to prepare cell suspensions at 2 × 10^6^ cells/mL. According to the manufacturer’s protocol, 10 μg/mL CD62P (recombinant human P-selectin) (R&D, MINN, USA) and 2 μg/mL CD62E (recombinant human E-selectin) (R&D, MINN, USA) were added to each 100 μL of cell suspension, and cells were then incubated for 1 h at 37 °C in the dark. Another 5 μL of CD62E-PE and 5 μL of CD62P-APC were added to each group of cells, followed by incubation at 4 °C in the dark for 30 min. The cells were washed twice using centrifugation and analyzed on a flow cytometer.

The passage P3 glycosylated BMSCs and untreated BMSCs were cultured for two weeks in the osteogenic induction medium and then stained with Alizarin Red. The number of calcified nodules in random visual fields was determined using the microscope and then compared between these two groups to evaluate their osteogenic potential.

The passage P3 glycosylated BMSCs and untreated BMSCs were cultured for two weeks in the osteogenic induction medium (BMSCs cultured without osteogenic induction served as the negative control group). Total RNA was extracted from all three groups of BMSCs, using the same RNA extraction method as described previously. Reverse transcription and PCR were also performed as described previously. The primer sequences used are listed in Table 3.

**Table 3:** Primer sequences (5'-3') used for RT-PCR

Gene	Forward	Reverse
Col I	GCTCCTCTTAGGGGCCACT	ATTGGGGACCCTTAGGCCAT
Runx2	GACTGTGGTTACCGTCATGGC	ACTTGGTTTTTCATAACAGCGGA
GAPDH	AGGTCGGTGTGAACGGATTTG	GGGGTCGTTGATGGCAACA

### Detection of the directional homing ability of glycosylated BMSCs in mid-femoral bone defect mouse model

SPF FVB/N male mice (8-week-old) were injected intraperitoneally with 4% chloral hydrate for anesthesia. Next, the right lower limb skin was prepared and disinfected. The skin over the middle section of the right thigh was cut open (~ 0.5 cm) and the thigh muscles were bluntly separated, exposing the middle section of the femur. After expanding the surrounding muscles, a dental micro drill with a 1.0-mm drill bit was used to drill through the cortical bone on both sides of the femur perpendicularly. After the bone defect was successfully introduced, local disinfection was performed, and the incision was then closed layer by layer.

Twelve FVB/N mice were subjected to the same bone defect procedures as described above. The mice were divided into the FuT7-BMSCs group and the Vector-BMSCs group (n = 6/group). Green fluorescence was observed from the cells in both groups. The cell concentration was adjusted to 1 × 10^7^ cells/mL. A 1-mL syringe was used to aspirate the cell suspension and inject it into the mouse through the tail vein. Mice were injected with 300 μL of the cell suspension per mouse and then sacrificed at 6 and 12 h. The bone section 0.5 cm above and below the right femoral defect was dissected out and both ends of the bone were cut off. A syringe containing 1 mL of the DMEM/F12 complete medium was used to wash the retained diaphysis repeatedly. The washed-out cells were plated in a 24-well plate and placed into a cell culture incubator for 1 h. After 1 h, the culture plate was taken out from the incubator and placed under a fluorescence microscope for observation. Three random visual fields were selected from each well to image, count, and calculate the results.

The firefly luciferase control lentiviruses were used to transfect the FuT7-BMSCs and untreated BMSCs using the same approach as described above. After successful transfection, two groups of passage P2 cells were taken and the cell concentration was adjusted to 1 × 10^7^ cells/mL. Mice with femoral bone defects were injected through the tail vein (n = 6/group). Mice from the corresponding groups were taken at 6 h and 12 h after the surgery, and then injected intraperitoneally with D-firefly luciferin potassium salt (substrate) at 15 mg/mL, 10 μL/g, according to the instructions. After 20 min, in vivo imaging was performed to observe and analyze cell homing.

Thirty-six mice with bone defects (n = 12/group) were injected with FuT7-BMSCs and untreated BMSCs through the tail vein. The blank control group was injected with an equal volume of normal saline. At days 7 and 14, the femurs were dissected out from the defect location and analyzed by Micro-CT (PerkinElmer, USA). The defect area, Bone volume (BV)/Tissue volume (TV) ratio, and other parameters were determined.

Eighteen mice with bone defects (n = 6/group) were injected with FuT7-BMSCs and untreated BMSCs through the tail vein. The blank control group was injected with an equal volume of normal saline. On day 10, the femurs containing bone defects were removed, the soft tissues around the femurs were cleaned, and the femurs were then fixed in 4% paraformaldehyde for three days. The femurs were decalcified in the ethylene diamine tetraacetie acid (EDTA) solution (Solarbio, Beijing, China) for approximately 14 d, paraffin-embedded, and sectioned. Next, the sections were staining with H&E and Masson (Solarbio, Beijing, China) according to the assay kit instructions. The specimens were then observed and imaged under a microscope to evaluate the effect of different BMSCs on bone defect repair.

### Statistical analysis

The SPSS 20.0 (IBM, USA) statistical software was used for the statistical analysis. The experimental data were expressed as the mean ± standard deviation, and the data were analyzed using one-way analysis of variance (ANOVA). The *t*-test was used to perform the one-to-one comparison between the experimental and control groups. The difference was considered to be statistically significant at *p* < 0.05.

## Supplementary Information

**Additional file 1**: **Figure S1**. Schematic diagram of the glycosylated BMSCs migration experiment in vitro.

## Data Availability

Not applicable.

## Abbreviations

BMSCs: Bone marrow mesenchymal stem cells
sLe^X^: Sialyl Lewis-X
MSCs: Mesenchymal stem cells
HSPCs: Hematopoietic stem cells
IL-1: Interleukin-1
TNF-α: Tumor necrosis factor-alpha
FuT7: Fucosyltransferase VII
GFP: Green fluorescence
