# Supplementary figures and images for: Directional homing of glycosylation-modified bone marrow mesenchymal stem cells for bone defect repair
Source: J Nanobiotechnology. 2021 Jul 31;19:228. doi: 10.1186/s12951-021-00969-3 (PMC8325817; doi:10.1186/s12951-021-00969-3)

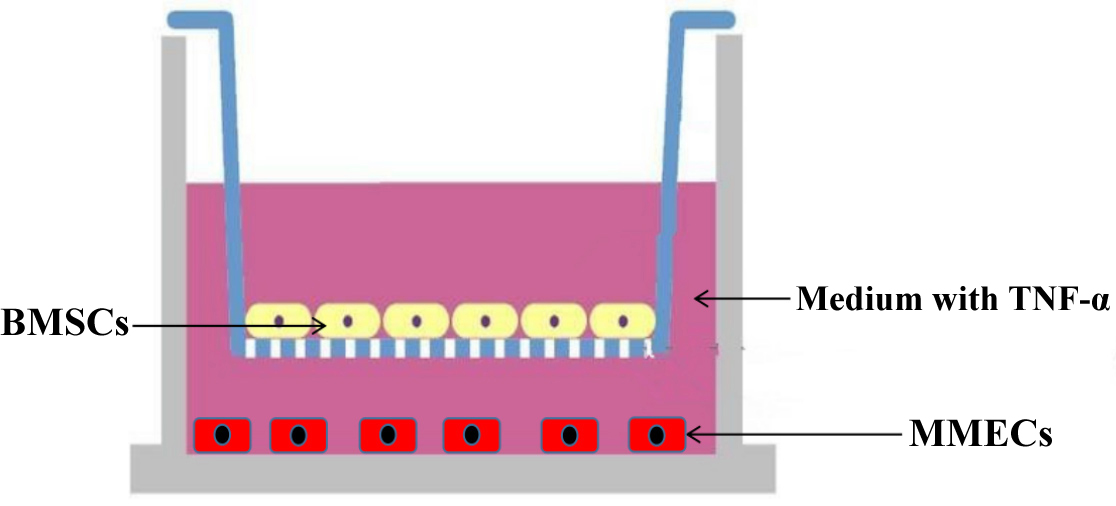

Supplement: Supplementary file 1 — Additional file 1: Figure S1. Schematic diagram of the glycosylated BMSCs migration experiment in vitro. [file 12951_2021_969_MOESM1_ESM.jpg]
